# Cache and energy efficient algorithms for Nussinov’s RNA Folding

**DOI:** 10.1186/s12859-017-1917-0

**Published:** 2017-12-06

**Authors:** Chunchun Zhao, Sartaj Sahni

**Affiliations:** Department of Computer and Information Science and Engineering, University of Florida, Gainesville, 32611 FL USA

**Keywords:** RNA Folding, Nussinov’s algorithm, Cache efficient

## Abstract

**Background:**

An RNA folding/RNA secondary structure prediction algorithm determines the non-nested/pseudoknot-free structure by maximizing the number of complementary base pairs and minimizing the energy. Several implementations of Nussinov’s classical RNA folding algorithm have been proposed. Our focus is to obtain run time and energy efficiency by reducing the number of cache misses.

**Results:**

Three cache-efficient algorithms, *ByRow*, *ByRowSegment* and *ByBox*, for Nussinov’s RNA folding are developed. Using a simple LRU cache model, we show that the *Classical* algorithm of Nussinov has the highest number of cache misses followed by the algorithms *Transpose* (Li et al.), *ByRow*, *ByRowSegment*, and *ByBox* (in this order). Extensive experiments conducted on four computational platforms–Xeon E5, AMD Athlon 64 X2, Intel I7 and PowerPC A2–using two programming languages–C and Java–show that our cache efficient algorithms are also efficient in terms of run time and energy.

**Conclusion:**

Our benchmarking shows that, depending on the computational platform and programming language, either *ByRow* or *ByBox* give best run time and energy performance. The C version of these algorithms reduce run time by as much as 97.2*%* and energy consumption by as much as 88.8*%* relative to *Classical* and by as much as 56.3*%* and 57.8*%* relative to *Transpose*. The Java versions reduce run time by as much as 98.3*%* relative to *Classical* and by as much as 75.2*%* relative to *Transpose*. *Transpose* achieves run time and energy efficiency at the expense of memory as it takes twice the memory required by *Classical*. The memory required by *ByRow*, *ByRowSegment*, and *ByBox* is the same as that of *Classical*. As a result, using the same amount of memory, the algorithms proposed by us can solve problems up to 40% larger than those solvable by *Transpose*.

## Background

### Introduction

RNA secondary structure prediction (i.e., RNA folding) [1] *“is the process by which a linear ribonucleic acid (RNA) molecule acquires secondary structure through intra-molecular interactions. The folded domains of RNA molecules are often the sites of specific interactions with proteins in forming RNA–protein (ribonucleoprotein) complexes.”* Unlike a paired double strand DNA sequence, RNA primary structure is single strand which could be considered as a chain (sequence format) of nucleotides, where the alphabet is {A (adenine), U(uracil), G(guanine), C(cytosine)}. This single strand could fold onto itself such that (A, U), (C, G) and (G, U) are complementary base pairs. The secondary structure of RNA is such two-dimensional structure composed by list of complementary base pairs which are close together with the minimum energy. RNA folding algorithm is the approach to predict this secondary structure of RNA. In other words, we are given a primary structure of RNA, which is a list of sequence characters *A*[ 1:*n*]=*a*_1_ *a*_2_⋯*a*_*n*_ where *a*_*i*_∈*A,U,G,C*. We are required to determine this non-nested/pseudoknot-free structure P with minimum energy, such that the number of complementary base pairs in P is maximum. (A pseudoknot [2] *“is a nucleic acid secondary structure containing at least two stem-loop structures in which half of one stem is intercalated between the two halves of another stem.”*)

Smith and Waterman (SW) [3] and Nussinov et al. [4] proposed a dynamic programming algorithm for RNA folding in 1978. Zuker et al. [5] modified Nussinov’s algorithm using thermodynamic and auxiliary information. The asymptotic complexity of the SW’s, Nussinov’s, and Zuker’s algorithms are *O*(*n* ^3^) time and *O*(*n* ^2^) space, where *n* is the length of the RNA sequence. Li et al. [6] proposed a cache-aware version of Nussinov’s algorithm, called *Transpose*, that takes twice the memory but reduces run time significantly. Many parallel algorithms for RNA folding have also been proposed (see, for e.g., [6–15]).

In this paper, we focus on reducing the number of cache misses that occur in the computation of Nussinov’s method without increasing the memory requirement. Our interest in cache misses stems from two observations–(1) the time required to service a lowest-level-cache (LLC) miss is typically 2 to 3 orders of magnitude more than the time for an arithmetic operation and (2) the energy required to fetch data from main memory is typical between 60 to 600 times that needed when the data is on the chip. As a result of observation (1), cache misses dominate the overall run time of applications for which the hardware/software cache prefetch modules on the target computer are ineffective in predicting future cache misses. The effectiveness of hardware/software cache prefetch mechanisms varies with application, computer architecture, compiler, and compiler options used. So, if we are writing code that is to be used on a variety of computer platforms, it is desirable to write cache-efficient code rather than to rely exclusively on the cache prefetching of the target platform. Even when the hardware/software prefetch mechanism of the target platform is very effective in hiding memory latency, observation (2) implies excessive energy use when there are many cache misses.

We develop three algorithms that meet our objective of cache efficiency without memory increase–*ByRow*, *ByRowSegment*, and *ByBox*. Since these take the same amount of memory as *Classical* and *Transpose* takes twice as much, the maximum problem size (*n*) that can be solved in any fixed amount of memory by algorithms *Classical*, *ByRow*, *ByRowSegment*, and *ByBox* is 40% more than what can be done by *Transpose*. On practical but large instances, *ByRow* and *ByRowSegment* have the same run time performance. Our experiments indicate that, depending on the computational platform and programming language, either *ByRow* or *ByBox* give best run time and energy performance. In fact, the C version of our proposed algorithms reduce run time by as much as 97.2*%* and energy consumption by as much as 88.8*%* relative to *Classical* and by as much as 56.3*%* and 57.8*%* relative to *Transpose*. The Java versions reduce run time by as much as 98.3% relative to *Classical* and by as much as 75.2% relative to *Transpose*.

The rest of the paper is organized in the following way. We first introduce our simple cache model that we use in our cache-efficiency analysis. Then we propose three cache- and memory-efficient RNA folding algorithms. These algorithms are being theoretically analyzed using our cache model. Finally, we present our experimental and benchmark results.

### Cache model

We use a simple cache model so that the cache miss analysis is of manageable complexity. In this model, there is a single cache whose capacity is *sw* words, where *s* is the number of cache lines and *w* is the number of words in a cache line. Each data item is assumed to have the same size as a word. The main memory is assumed to be partitioned into blocks of size *w* words each. Data transfer between the cache and memory takes place in units of a block (equivalently, a cache line). A read miss occurs whenever the program attempts to read a word that is not in cache. To service this cache miss, the block of main memory that includes the needed word is fetched and copied into a cache line, which is selected using the LRU (least recently used) rule. Until this block of main memory is evicted from this cache line, its words may be read without additional cache misses. We assume the cache is written back with write allocate. That is, when the program needs to write a word of data, a write miss occurs if the block corresponding to the main memory is not currently in cache. To service the write miss, the corresponding block of main memory is fetched and copied in a cache line. Write back means that the word is written to the appropriate cache line only. A cache line with changed content is written back to the main memory when it is about to be overwritten by a new block from main memory.

In practice, modern computers commonly have two or three levels of cache and employ sophisticated adaptive cache replacement strategies rather than the LRU strategy described above. Further, hardware and software cache prefetch mechanisms, out of order executions are often deployed to hide the latency involved in servicing a cache miss. These mechanisms may, for example, attempt to learn the memory access pattern of the current application and then predict the future need for blocks of main memory. The predicted blocks are brought into cache before the program actually tries to read/write from/into those blocks thereby avoiding (or reducing) the delay involved in servicing a cache miss. Actual performance is also influenced by the compiler used and the compiler options in effect at the time of compilation.

As a result, actual performance may bear little relationship to the analytical results obtained for our simple cache model. Despite this, we believe the simple cache model serves a useful purpose in directing the quest for cache-efficient algorithms that eventually need to be validated experimentally. We believe this because our simple model favors algorithms that exhibit good spatial locality in their data access pattern over those that do not and all cache architectures favor algorithms with good spatial locality. The experimental results reported in this paper strengthen our belief in the usefulness of our simple model. These results indicate that algorithms with a smaller number of cache misses on our simple model actually have a smaller number of (lowest level) cache misses on a variety of modern computers that employ potentially different cache replacement strategies (vendors often use proprietary cache replacement strategies). Further, a reduction in cache misses on our simple model often translates into a reduction in run time.

## Methods

### *Classical* RNA folding algorithm (Nussinov’s algorithm)

**Fig. 1:**
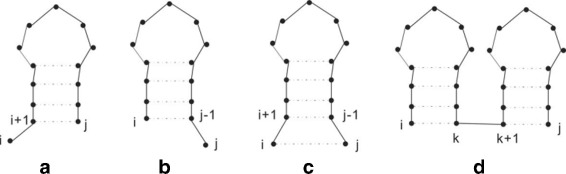
Four cases for Nussinov’s equations [21]

Let *A*[ 1:*n*]=*a*_1_ *a*_2_⋯*a*_*n*_ be an RNA sequence and let *H*_*ij*_ be the maximum number of the complimentary pairs in a folding of the sub-sequence *A*[ *i*:*j*], 1≤*i*≤*j*≤*n*. So, *H*_1*n*_ is the score of the best folding for the entire sequence *A*[ 1:*n*]. The following dynamic programming equations to compute *H*_1*n*_ are due to Nussinov [4].

1 $$ H_{i,i-1} = 0, \ 2 \le i \le n  $$

2 $$ H_{i,i} = 0, \ 1 \le i \le n  $$

3 $$ H_{i,j} = \text{max}\left\{ \begin{array} {lcr} H_{i+1,j} \\ H_{i,j-1} \\ H_{i+1,j-1} + c(a_{i}, a_{j}) \\ {max}_{i<k<j}\{H_{i,k}+ H_{k+1,j}\} \end{array} \right.  $$

where *c*(*a*_*i*_,*a*_*j*_) is the match score between characters *a*_*i*_ and *a*_*j*_. If *a*_*i*_ and *a*_*j*_ are complimentary pairs such as *AU*, *GC* or *GU*, *c*(*a*_*i*_,*a*_*j*_) is 1, otherwise it is 0. The different cases of the recurrence in Nussinov’s algorithm are illustrated in Fig. 1, where Fig. 1 a shows the case when *a*_*i*_ is added to the best RNA folding of the subsequence *A*[ *i*+1:*j*]. Figure 1 b shows the case when *a*_*j*_ is added to the best RNA folding of *A*[ *i*:*j*−1], Fig. 1 c shows the case when (*a*_*i*_,*a*_*j*_) is added to the best RNA folding of *A*[ *i*+1:*j*−1] and Fig. 1 d shows the combining of two subsequences *A*[ *i*:*k*] and *A*[ *k*+1:*j*] into one.

Due to the fact that Fig. 1 a and b can be considered as a special case of combining two subsequences where one of them is a single node subsequence. Several authors ([15], for example) have observed that Nussinov’s equations may be simplified to

4 $$ H_{i,i} = 0, \ 1 \le i \le n  $$

5 $$ H_{i,i+1} = 0, \ 1 \le i \le n-1  $$

6 $$ H_{i,j} = \text{max}\left\{ \begin{array} {lcr} H_{i+1,j-1} + c(a_{i}, a_{j}) \\ {max}_{i\le{k}<j}\{H_{i,k}+ H_{k+1,j}\} \end{array} \right.  $$

Once the best RNA folding score, *H*_1*n*_, has been computed, a standard dynamic programming traceback procedure, which takes *O*(*n*) time, may be performed to find the path leading to the maximum score. This path defines the actual RNA secondary structure.

Algorithm 1 gives the *Classical* algorithm to compute *H*_1*n*_ using the simplified Nussinov’s equations. This algorithm computes *H* by diagonals and within a diagonal from top to bottom. It’s run time is *O*(*n* ^3^). Although the algorithm is written using two-dimensional array notation for *H*, we need only the upper triangle of *H*. Hence, a memory efficient implementation would either map the upper triangle into a 1D array or employ a dynamically allocated 2D array with variable size rows. In either case, we would need memory for *n*(*n*+1)/2 elements of *H* rather than for *n* ^2^ elements.



**Fig. 2:**
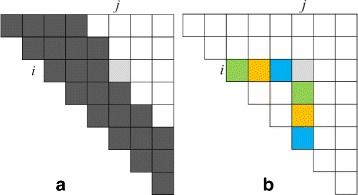
Memory access pattern for algorithm *Classical* (Algorithm 1)

For the (data) cache miss analysis, we focus on read and write misses of the array *H* and ignore misses due to the reads of the sequence *A* as well as of the scoring matrix *c* (notice that there are no write misses for *A* and *c*). Figure 2 shows the memory access pattern for *H*. Figure 2 a left shows the order (by diagonals and within a diagonal from top to bottom) in which the elements of *H* are computed. In this figure, three diagonals have been computed as have 2 elements of the fourth; we are presently computing the third element (*H*_*ij*_) of the fourth diagonal. Figure 2 b shows the elements of *H* in row *i* and column *j* that are needed for the computation of *H*_*ij*_ (i.e., in the computation of max{*H*_*i,k*_+*H*_*k*+1,*j*_}). The elements in row *i* are accessed from left to right while those in column *j* are accessed from top to bottom. So, *w* row elements are brought into cache with a single miss and a miss takes place for each element of column *j* that is accessed. Note that the cache lines for column *j* also contain the column *j*+1 data needed in the computation of *H*_*i*+1,*j*+1_. However, when *n* is sufficiently large, this data is overwritten by new data under the LRU policy before it can be used in the computation of *H*_*i*+1,*j*+1_. So, for each of the *j*−*i* sums of max{*H*_*i,k*_+*H*_*k*+1,*j*_} we incur 1/*w* read misses on average for *H*_*i,k*_ and 1 read miss for *H*_*k*+1,*j*_. Over the entire computation we compute *n* ^3^/6 (plus low order terms) of these sums incurring a total of (*n* ^3^/6)(1+1/*w*) read misses. Although to complete the computation of *H*_*i,j*_ we also need *H*_*i*+1,*j*−1_, accessing these values of *H* incurs only *O*(*n* ^2^) read misses. The number of write misses for *H* is also *O*(*n* ^2^). So, for our simplified cache model, the number of cache misses incurred when computing *H* using algorithm *Classical* is (*n* ^3^/6)(1+1/*w*) (plus low order terms).

### *Transpose* RNA folding algorithm

Li et al. [6] have proposed a cache-efficient computation of Nussinov’s simplified equations. Their algorithm, which we refer to as *Transpose*, uses an *n*×*n* array *H* in which the upper triangle is used to store the *H*_*i,j*_, *j*≤*i*, values defined by Nussinov’s equations and the lower triangle is used to store the transpose of the upper triangle. That is, *H*_*i,j*_=*H*_*j,i*_ for all *i* and *j*. As new *H*_*ij*_s are computed, they are stored in both *H*_*i,j*_ and *H*_*j,i*_. The sum *H*_*i,k*_+*H*_*k*+1,*j*_ is computed as *H*_*i,k*_+*H*_*j,k*+1_, with the result that a sum now requires only 2/*w* cache misses on average. So, the total number of read misses is (*n* ^3^/6)(2/*w*) plus low order terms. The number of write misses is *O*(*n* ^2^). The ratio of cache misses of *Classical* to *Transpose* is approximately (1+1/*w*)/(2/*w*)=(*w*+1)/2. The run time remains *O*(*n* ^3^).

### *ByRow* RNA folding algorithm

Although *Transpose* reduces the number of cache misses (in our model) by an impressive factor of (*w*+1)/2 relative to *Classical*, it does so at the cost of doubling the memory requirement. The increased memory requirement means that *Classical* can be used to solve problems up to 40% bigger than can be solved by *Transpose* on any computer with a fixed memory size. For smaller instances that can be solved by both algorithms, we expect *Transpose* to take less time. In this section, we propose an alternative cache-efficient algorithm *ByRow* that does not have a memory penalty associated with it. In our cache model, *ByRow* incurs the same number of cache misses as incurred by *Transpose*.

**Fig. 3:**
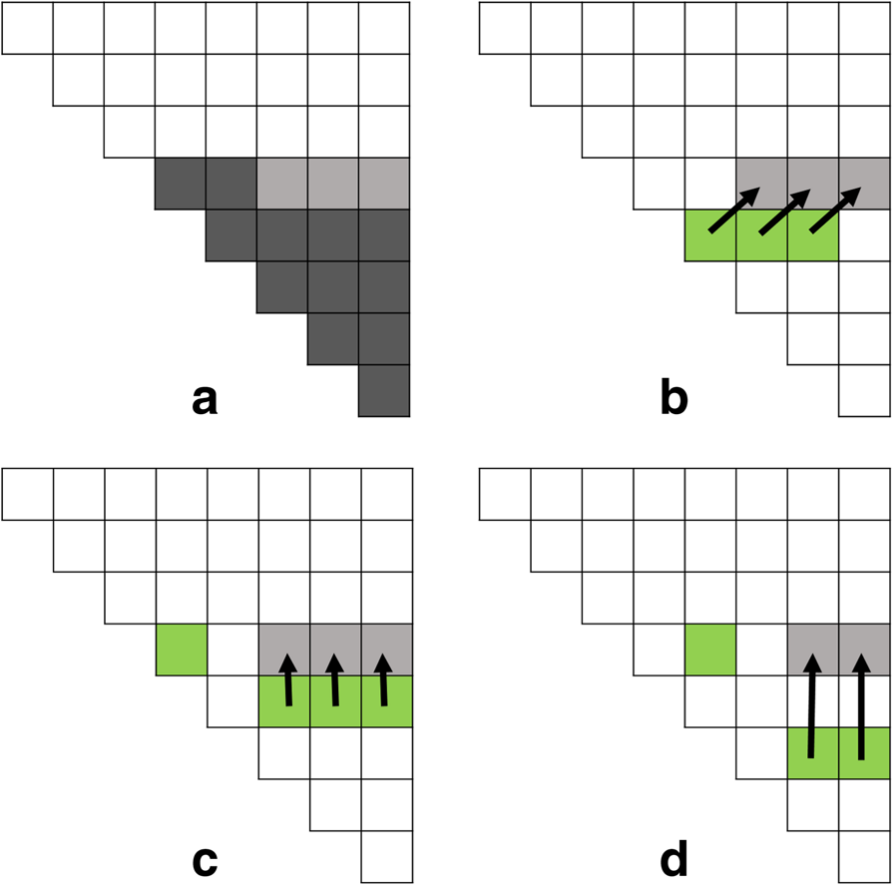
Memory access pattern for *ByRow* algorithm (Algorithm 2)



The algorithm *ByRow* computes the *H*_*i,j*_ *s* by row bottom-to-top and within a row left-to-right. This is illustrated in Fig. 3. Figure 3 a shows the situation after the 4 bottommost rows of *H* have been computed. The computation of the next row (i.e, row 5 from the bottom in our example) is done in two stages. Note that the first two elements on each row are 0 by definition. So, only elements 3 onward are to be computed. In the first stage, every *H*_*i,j*_, *j*>*i*+1 on the row being computed is initialized to *H*_*i*+1,*j*−1_. The memory access pattern for this is shown in Fig. 3 b. The second stage comprises many sub-stages. In a sub-stage, all *H*_*i,j*_s in row *i* are updated using the sums *H*_*i,k*_+*H*_*k*+1,*j*_ for a single *k*. In the first sub-stage, we use *H*_*i,i*_ and *H*_*i*+1,*j*_ to update *H*_*i,j*_, *j*>*i*+1 (see Fig. 3 c). In the next sub-stage, we use *H*_*i,i*+1_ and *H*_*i*+1,*j*_ to update *H*_*i,j*_, *j*>*i*+1 and so on. Algorithm 2 gives the details.

It is easy to see that *ByRow* takes *O*(*n* ^3^) time and that its memory requirement is the same as that of *Classical* and about half that of *Transpose*. For the cache miss analysis, we see that for each element initialized in stage 1, an average of 1/*w* read misses and 1/*w* write misses occur. So, this stage contributes *O*(*n* ^2^) to the overall cache miss count. For the second stage, we see that the total number of read misses for the first term in an *H*_*i,k*_+*H*_*k*+1,*j*_ over all sub-stages is *O*(*n* ^2^/*w*) and that for the second term is (*n* ^3^/6)(1/*w*) (plus low order terms). Additionally, there are (*n* ^3^/6)(1/*w*) (plus low order terms) read misses for *H*_*i,j*_. So, the total number of misses is (*n* ^3^/6)(2/*w*) (plus low order terms).

The algorithm *ByRowSegment* reduces this count by computing the elements in each row of *H* in segments of size no larger than the capacity of our cache. The segments in a row are computed from left to right. When the segment size is *s*, the number of read misses for *H*_*ik*_ becomes (*n* ^3^/6)(1/*s*). The misses for *H*_*k*+1,*j*_ remains (*n* ^3^/6)(1/*w*). So, the total number of misses is further reduced to (*n* ^3^/6)(1/*s*+1/*w*).

### *ByBox* RNA folding algorithm

**Fig. 4:**
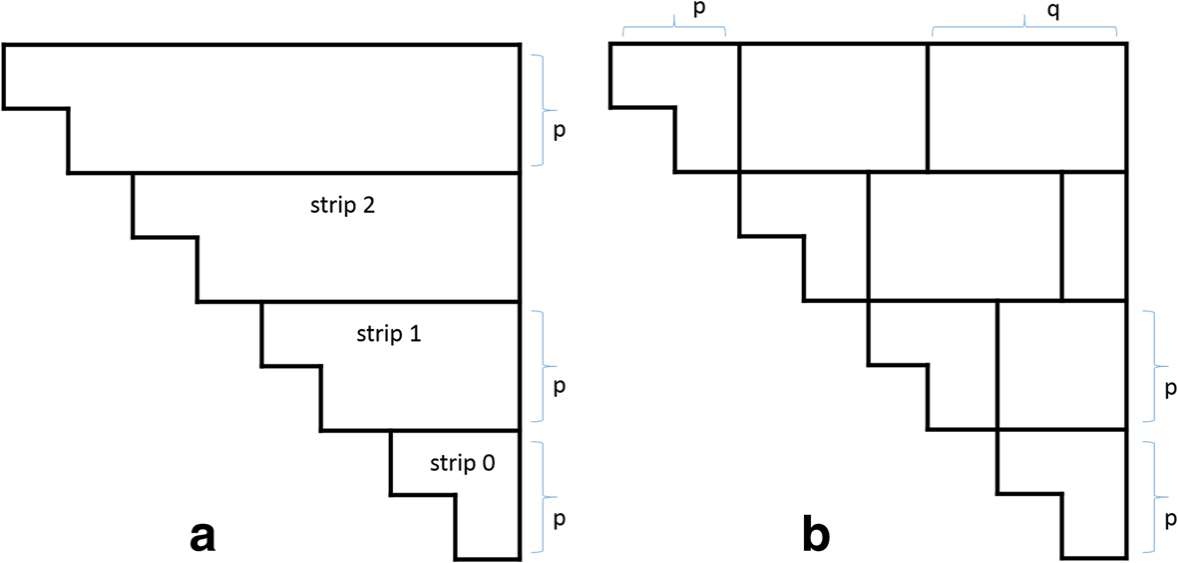
Partitioning *H* into boxes

In the *ByBox* algorithm, we partition *H* into boxes and compute these boxes in an appropriate order. For the partitioning, we first divide the rows of *H* into strips of *p* rows each from bottom-to-top (Fig. 4 a). Note that the top most strip may have fewer than *p* rows. Next each strip is partitioned into a triangle box and multiple rectangle boxes (Fig. 4 b). The width of the first box is *p*, that of all but the last of the remaining boxes is *q*, and that of the last is ≤*q*. Observe that the first box in a strip is a *p*×*p* triangle (the height of the triangle in the topmost strip may be less than *p*), the last box in a strip is a *p*×*q* rectangle (again the height in the top strip may be less than *p*), and the remaining boxes are *p*×*q* boxes (again, the height may be less in the top strip).

**Fig. 5:**
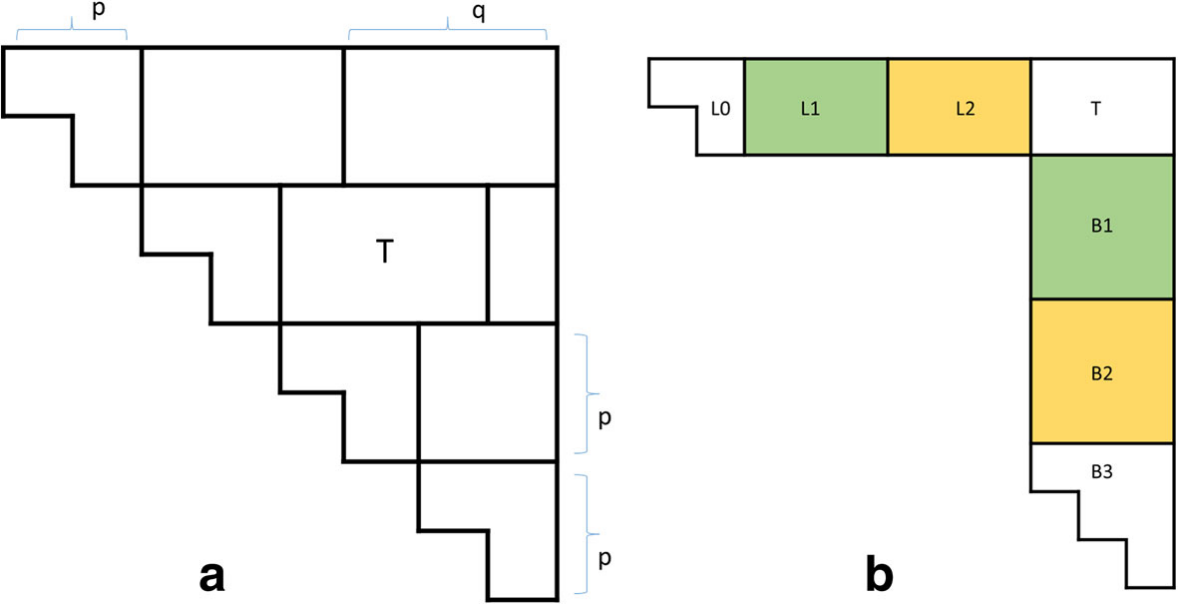
Boxes in the computation of the rectangular box *T*

The elements in triangular boxes are computed using *ByRow*. These triangular boxes may be computed in any order. The rectangular boxes are computed by strips bottom-to-top and within a strip from left-to-right. Let *T* denote the rectangular box to be computed next (Fig. 5 a). Because of the order in which rectangular boxes are computed, all *H* values to its left and below it have already been computed. Let *L*_0_, *L*_1_, ⋯, *L*_*k*−1_ be the boxes to the left of *T*. Note that *L*_0_ is a triangular box. Partition the *H*s below *T* into *q*×*q* boxes *B*_1_, *B*_2_, ⋯, *B*_*k*−1_ plus a last triangular box *B*_*k*_ whose width is *w* (Fig. 5 b).

To compute *T*, we first consider the pairs of rectangular boxes (*L*_*i*_,*B*_*i*_), 1≤*i*<*k*. When a pair (*L*_*i*_,*B*_*i*_) is considered, we update all *H*s in the box *T* that depend on values in this pair of boxes. To complete the computation of the *H*s in box *T*, we read in the triangular boxes *L*_0_ and *B*_*k*_ and update all *H*s in *T* by moving up the rows of *T* and within a row of *T* from left-to-right (Algorithm 3).



The time and memory required by algorithm *ByBox* are the same as for *Classical* and *ByRow*. For the cache miss analysis, assume that we have enough cache to hold one pair (*L*_*i*_,*B*_*i*_) as well as the box *T*. Loading *L*_*i*_ and *B*_*i*_ into cache incurs *pq*/*w* misses for *L*_*i*_ and *q* ^2^/*w* for *B*_*i*_. The number of *H*_*i,k*_+*H*_*k*+1,*j*_ computations we can do for each *H* in *T* without additional misses is *q*. So, with (*p*+*q*)*q*/*w* cache misses we can do *pq* ^2^ sum computations. Or, an average of (*p*+*q*)*q*/(*wpq* ^2^)=(*p*+*q*)/(*wpq*) misses per computation. Therefore, to do all *n* ^3^/6 required computations we incur (*n* ^3^/6)(*p*+*q*)/(*wpq*) cache misses. The misses attributable to the remaining terms in Nussinov’s equations as well as to writes of *H* are *O*(*n* ^2^) and may be ignored.

When *q*=*w*, the cache miss count for *ByBox* becomes (*n* ^3^/6)(1/*w* ^2^+1/(*wp*)), which is quite a bit less than that for our other algorithms.

When *p*=1, *ByBox* has much similarity with *ByRowSegment*. However, since *ByBox* needs sufficient cache for a *q*×*qB*_*i*_, $q \le \sqrt {s}$, where *s* is the largest segment size that can be accomodated in cache. So, the miss count for *ByBox* is $(n^{3}/6)(p+q)/(wpq) = (n^{3}/6)(1+1/\sqrt {s})(1/w)$, which is more than that for *ByRowSegment* when $w < \sqrt {s}$.

### Practical considerations

We make the following observations regarding our expectations for the performance of the various Nussinov’s algorithms described in this section:

1. We have used a very simple 1-level cache model for our analyses and also assumed an LRU replacement strategy. Modern computers have two or three levels of cache and employ more sophisticated cache replacement strategies. So, our analyses, are at best a crude approximation of actual cache misses.
2. Modern computers employ sophisticated hardware and software methods for cache miss prediction and prefetch data based on this prediction. To the extent these methods are successful in accurately predicting the need for data sufficiently in advance, the latency due to cache misses can be masked. As a result, observed run times may not be indicative of cache misses.
3. In practice, the maximum *n* will be small enough that many of the cache misses counted in our analyses will actually not occur. For example, in the *ByRow* algorithm, the lowest level cache will usually be large enough to hold a row of *H*. This expectation comes from the observation that when *n*=100,000 (say), we will need more than 2×10^10^ bytes of main memory to hold the upper triangle of *H* (assuming 4 bytes per element) and only 400,000 bytes of cache to hold a row of *H*. As a result, the cache misses for *H*_*i,j*_ will be *O*(*n* ^2^) rather than *O*(*n* ^3^). Similarly, for *ByRowSegment*, *s*=*n*. So, in practice, we expect *ByRow* and *ByRowSegment* to have the same performance.
4. In *ByBox*, using a *q* as small as *w* is not expected to result in speedup because of the overheads involved in this algorithm. In practice, we wish to use large nearly square boxes such that *L*_*i*_, *B*_*i*_, and *T* fit in cache. When the size of the lowest level cache is sufficient for 3∗2^20^ elements (say), we could set *p*=*q*=1024.

## Results

### Experimental platform and test data

We implemented the *Classical*, *Transpose*, *ByRow*, and *ByBox* RNA folding algorithms in two programming languages – C and Java. For the data set sizes used by us, *ByRow* and *ByRowSegment* are identical as a row fits into cache and the segment size equals the row size. Consequently, we did not experiment with *ByRowSegment*. For all but *Transpose*, we conducted preliminary tests benchmarking 3 different implementations as below:

1. *H* is a classical *n*×*n* array.
2. The upper triangle of *H* is mapped into a 1D array of size *n*(*n*+1)/2 in row-major order [16].
3. *H* is a 2D array with variable size rows. The first row has *n* entries, the next has *n*−1, the next has *n*−2, ⋯ and the last has 1 entry. Such an array may be dynamically allocated as in [16]

The last two of these implementations take about half the memory as taken by *Transpose* and the first implementation. Our preliminary benchmarking showed that, in C, the last implementation is faster than the other two while in Java the first implementation is the fastest and the third next fastest. More specifically, the third implementation takes between 1% and 4% less time than the first in C and approximately 10% more time than the first in Java. The performance results reported in this section are for the third implementation except in the case of the smaller Java tests for which we had sufficient memory to use implementation 1. In other words, the reported performance results are for the fastest of the three possible implementations for *Classical*, *ByRow*, and *ByBox*. For *Transpose*, the standard 2D array implementation is used as this algorithm uses the entire *n*×*n* array.

The following platforms were used to compile and execute the codes.

1. Xeon E5-2603 v2 Quad Core processor 1.8 GHz with 10 MB cache On this platform, the C codes were compiled using gcc version 5.2.1 with the O2 option and the Java codes were compiled using javac version 1.8.0_72.
2. AMD Athlon 64 X2 5600+ 2.9 GHz with 512 KB LLC cache. The C codes were compiled using gcc version 4.9.2 with the O2 option and the Java codes were compiled using javac version 1.8.0_73.
3. Intel I7-x980 3.33 GHz CPU with 12 MB LLC cache. The C codes were compiled using gcc 4.8.4 with the O2 option and the Java codes were compiled using javac 1.8.0_77.
4. PowerPC A2 processor(IBM Blue Gene Q) 1.33 GHz 64-bit with 32 MB LLC cache. On this platform, the C codes were compiled using Mpixlc: IBM XL C/C++ for Blue Gene Version 12.01. The Java codes were not run on this platform.

Our Xeon platform had tools to measure cache misses and energy consumption. So, for this platform we report cache misses and energy consumption as well as run time. On this platform, we used the “perf” [17] software to measure energy usage through the RAPL interface. For the PowerPC A2 (Blue Gene Q) platform, the MonEQ software [18, 19] was used to measure the power usage every half second and calculate the actual energy consumption. For the remaining 2 platforms (Xeon and AMD), we were able to determine only the run time as we did not have the tools available to measure cache misses and energy.

For test data, we used randomly generated RNA sequences as well as real RNA sequences obtained from the National Center for Biotechnology Information (NCBI) database [20].

### C Implementations

#### Xeon E5-2603

**Fig. 6:**
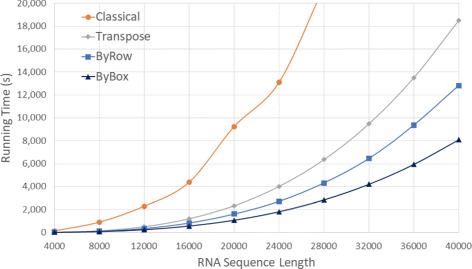
Run time, in seconds, for random sequences on Xeon E5 platform

**Fig. 7:**
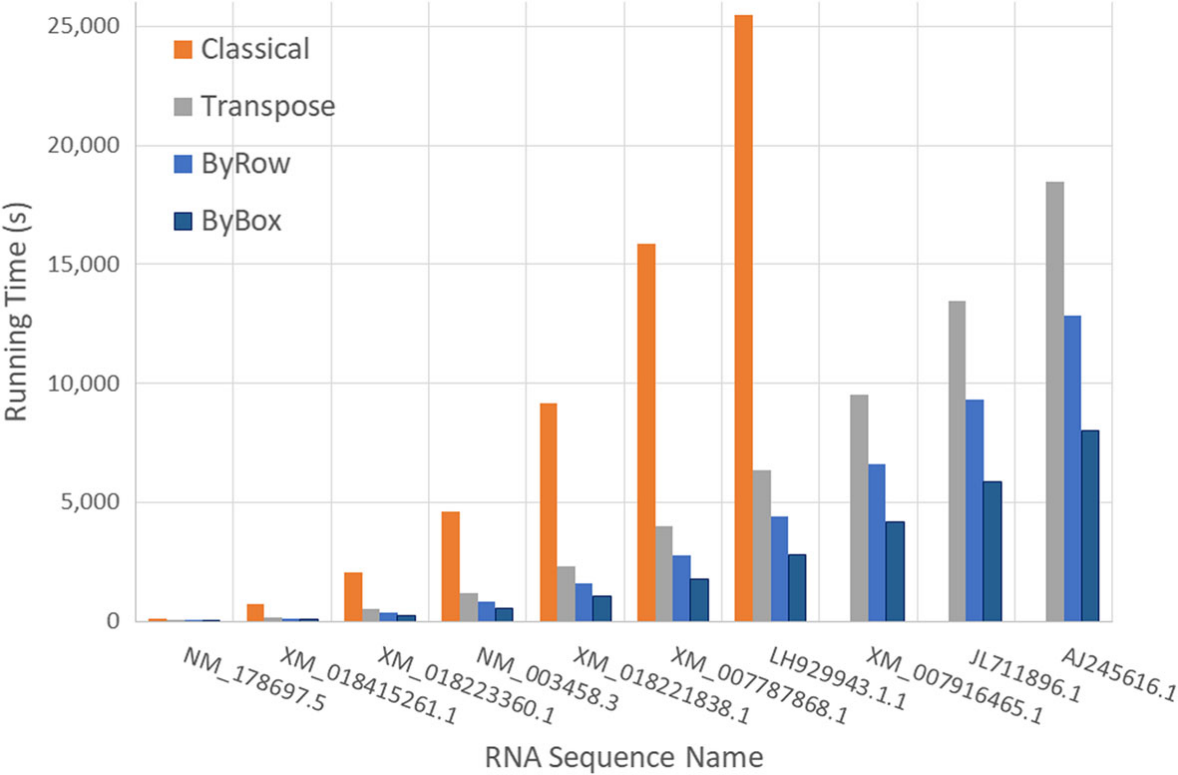
Run time, in seconds, for RNA sequences from [20] on Xeon E5 platform

Figure 6 and Table 1 give the run times of our various algorithms for our random data sets on our Xeon platform for sequence sizes between 4000 and 40000. Figure 7 and Table 2 do this for sample real RNA sequences from [20]. In both figures, the time is in seconds while in both tables, the time is given using the format *hh*:*mm*:*ss*. We did not measure the time required by *Classical* for *n*>28,000 as this algorithm took almost 6 hours for *n*=28,000. The column labeled *RvsC* (*BvsC*) in Tables 1 and 2 gives the run time reduction achieved by *ByRow* (*ByBox*) relative to *Classical*. Similarly, *RvsT* and *BvsT* give the reductions relative to *Transpose*. As can be seen, on our Xeon platform, *ByRow* performs better than *Classical* and *Transpose* algorithms, *ByBox* outperforms all other three algorithms. On the randomly generated data set, the *ByRow* algorithm reduces run time by up to 89.13*%* compared to the original Nussinov’s *Classical* algorithm and by up to 35.18*%* compared to the cache-efficient *Transpose* algorithm of Li et al. [6]. The corresponding reductions for *ByBox* are up to 91.26*%* and 56.31*%*. On the real RNA sequences, *ByRow* algorithm reduces run time by up to 90.38*%* and 35.19*%* compared to *Classical* and *Transpose* algorithm. The corresponding reductions for *ByBox* are up to 91.93*%* and 56.58*%*.

**Table 1:** Run time (HH:mm:ss) for random sequences on Xeon E5 platform

SeqLength	Classical	Transpose	ByRow	ByBox	RvsC	RvsT	BvsC	BvsT
4000	0:02:02	0:00:20	0:00:13	0:00:11	89.13%	35.18%	90.79%	45.03%
8000	0:14:49	0:02:35	0:01:44	0:01:18	88.30%	33.10%	91.26%	50.00%
12,000	0:37:51	0:08:33	0:05:51	0:04:05	84.55%	31.61%	89.20%	52.17%
16,000	1:12:49	0:20:08	0:13:47	0:09:22	81.08%	31.55%	87.14%	53.49%
20,000	2:33:39	0:38:54	0:26:48	0:17:49	82.56%	31.12%	88.41%	54.22%
24,000	3:38:09	1:07:00	0:45:15	0:30:10	79.26%	32.46%	86.17%	54.98%
28,000	5:57:03	1:46:00	1:12:01	0:47:18	79.83%	32.06%	86.75%	55.37%
32,000	-	2:37:50	1:47:28	1:09:58	-	31.91%	-	55.68%
36,000	-	3:44:51	2:35:58	1:38:50	-	30.63%	-	56.05%
40,000	-	5:08:10	3:33:43	2:14:38	-	30.65%	-	56.31%

**Table 2:** Run time (HH:mm:ss) for real RNA sequences of [20] on Xeon E5 platform

Code	SeqLength	Classical	Transpose	ByRow	ByBox	RvsC	RvsT	BvsC	BvsT
NM_178697.5	4008	0:02:17	0:00:20	0:00:13	0:00:11	90.38%	35.19%	91.93%	45.66%
XM_018415261.1	8011	0:11:56	0:02:36	0:01:44	0:01:17	85.45%	33.28%	89.30%	50.95%
XM_018223360.1	11,995	0:34:06	0:08:34	0:05:49	0:04:02	82.96%	32.16%	88.17%	52.92%
NM_003458.3	15,964	1:17:17	0:19:59	0:13:38	0:09:09	82.36%	31.77%	88.15%	54.18%
XM_018221838.1	19,957	2:32:50	0:38:39	0:26:36	0:17:25	82.60%	31.19%	88.61%	54.95%
XM_007787868.1	24,003	4:24:21	1:06:57	0:46:14	0:29:53	82.51%	30.94%	88.70%	55.37%
LH929943.1	28,029	7:04:35	1:46:18	1:13:34	0:46:59	82.67%	30.80%	88.93%	55.80%
XM_007916465.1	32,040	-	2:38:22	1:49:50	1:09:47	-	30.65%	-	55.93%
JL711896.1	35,962	-	3:44:10	2:35:14	1:37:39	-	30.75%	-	56.44%
AJ245616.1	40,003	-	5:08:02	3:34:06	2:13:44	-	30.49%	-	56.58%

Since the results for randomly generated RNA sequences are comparable to those for similarly sized sequences from the NCBI database [20], in the rest of paper, we present results only for randomly generated sequences.

**Fig. 8:**
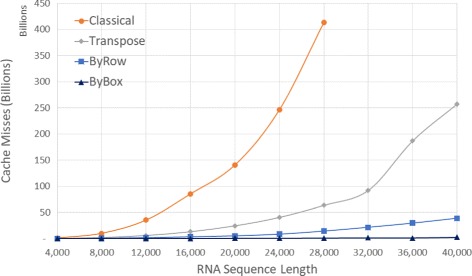
Cache Misses, in billions, for random sequences on Xeon E5 platform

Figure 8 and Table 3 gives the number of cache misses on our Xeon platform. *ByBox* reduces cache misses by up to 99.8*%* relative to *Classical* and by up to 99.3*%* relative to *Transpose*. The corresponding reductions for *ByRow* are 96.6*%* and 85.9*%*. The very significant reduction in cache misses is expected given the cache miss analysis was done using our simple cache model. The reduction in run time, while significant, isn’t as much as the reduction in cache misses possibly due to the effect of cache prefetching, which reduces cache induced computational delays.

**Table 3:** Cache misses, in millions, for random sequences on Xeon E5 server

SeqLength	Classical	Transpose	ByRow	ByBox	RvsC	RvsT	BvsC	BvsT
4,000	842	293	41	1	95.11%	85.94%	99.77%	99.35%
8,000	9,759	1,969	346	18	96.45%	82.39%	99.81%	99.07%
12,000	35,165	5,947	1,484	66	95.78%	75.04%	99.81%	98.89%
16,000	85,213	13,132	3,139	151	96.32%	76.10%	99.82%	98.84%
20,000	140,528	24,443	5,036	300	96.42%	79.39%	99.79%	98.77%
24,000	246,127	40,508	8,195	502	96.67%	79.77%	99.80%	98.76%
28,000	412,983	63,547	14,136	748	96.58%	77.75%	99.82%	98.82%
32,000	-	92,117	21,477	1,184	-	76.68%	-	98.71%
36,000	-	186,895	29,430	1,138	-	84.25%	-	99.39%
40,000	-	257,450	38,786	2,300	-	84.93%	-	99.11%

**Fig. 9:**
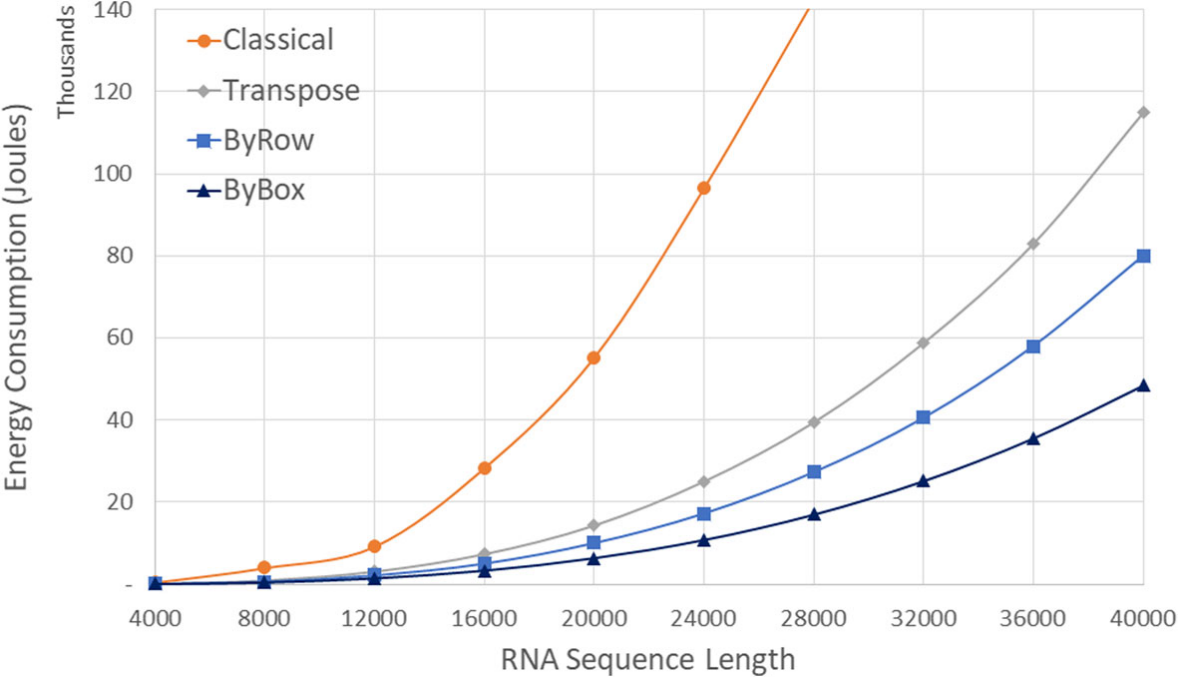
CPU and cache energy consumption, in thousands joules, for random sequences on Xeon E5 platform

Figure 9 and Tables 4 give the CPU and Cache energy consumption, in joules, by our Xeon platform. On our datasets, *ByBox* required up to 88.77*%* less CPU and Cache energy than *Classical* and up to 57.76*%* less than *Transpose*. It is interesting to note that the energy reduction is comparable to the reduction in run time suggesting a close relationship between run time and energy consumption for this application.

**Table 4:** CPU and cache energy consumption, in joules, for random sequences on Xeon E5 server

SeqLength	Classical	Transpose	ByRow	ByBox	RvsC	RvsT	BvsC	BvsT
4000	461.27	126.45	82.02	66.44	82.22%	35.14%	85.60%	47.46%
8000	4,009.37	957.73	649.48	461.25	83.80%	32.19%	88.50%	51.84%
12,000	9,191.74	3,164.37	2,184.09	1,462.51	76.24%	30.98%	84.09%	53.78%
16,000	28,291.57	7,405.47	5,074.94	3,357.95	82.06%	31.47%	88.13%	54.66%
20,000	55,183.94	14,374.95	10,053.49	6,395.33	81.78%	30.06%	88.41%	55.51%
24,000	96,430.95	25,082.80	17,254.61	10,825.72	82.11%	31.21%	88.77%	56.84%
28,000	142,359.14	39,491.70	27,332.57	17,004.35	80.80%	30.79%	88.06%	56.94%
32,000	-	58,821.30	40,551.20	25,204.38	-	31.06%	-	57.15%
36,000	-	82,974.06	58,011.66	35,620.84	-	30.08%	-	57.07%
40,000	-	114,886.41	80,002.00	48,531.81	-	30.36%	-	57.76%

#### AMD Athlon 64

**Fig. 10:**
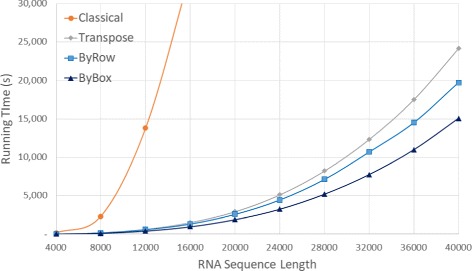
Run time, in seconds, for random sequences on AMD Athlon 64 server

Figure 10 and Table 5 give the run times on our AMD platform. The *Classical* algorithm took over 9 hours for *n*=16,000. As a result, we did not measure the run time of this algorithm for larger values of *n*. *ByBox* is faster than *ByRow* and both are substantially faster than *Classical* and *Transpose*. *ByBox* reduced run time by up to 97.16*%* compared to *Classical* and by up to 39.55*%* compared to *Transpose*. The reductions achieved by *ByRow* relative to *Classical* and *Transpose* were up to 96.08*%* and up to 18.33*%*, respectively.

**Table 5:** Run time, in HH:mm:ss, for random sequences on AMD Athlon 64 platform

SeqLength	Classical	Transpose	ByRow	ByBox	RvsC	RvsT	BvsC	BvsT
4000	0:02:35	0:00:25	0:00:22	0:00:15	85.97%	13.31%	90.22%	39.55%
8000	0:38:38	0:03:13	0:02:49	0:02:01	92.71%	12.35%	94.79%	37.35%
12,000	3:50:04	0:10:41	0:09:56	0:06:48	95.68%	7.11%	97.04%	36.35%
16,000	9:26:30	0:25:19	0:22:12	0:16:05	96.08%	12.35%	97.16%	36.46%
20,000	-	0:48:44	0:43:08	0:31:27	-	11.50%	-	35.46%
24,000	-	1:25:19	1:14:14	0:54:04	-	12.99%	-	36.61%
28,000	-	2:16:54	1:58:40	1:26:35	-	13.32%	-	36.75%
32,000	-	3:25:21	2:58:02	2:09:05	-	13.30%	-	37.14%
36,000	-	4:51:36	4:01:38	3:02:53	-	17.13%	-	37.28%
40,000	-	6:42:24	5:28:38	4:10:53	-	18.33%	-	37.65%

#### Intel I7

**Fig. 11:**
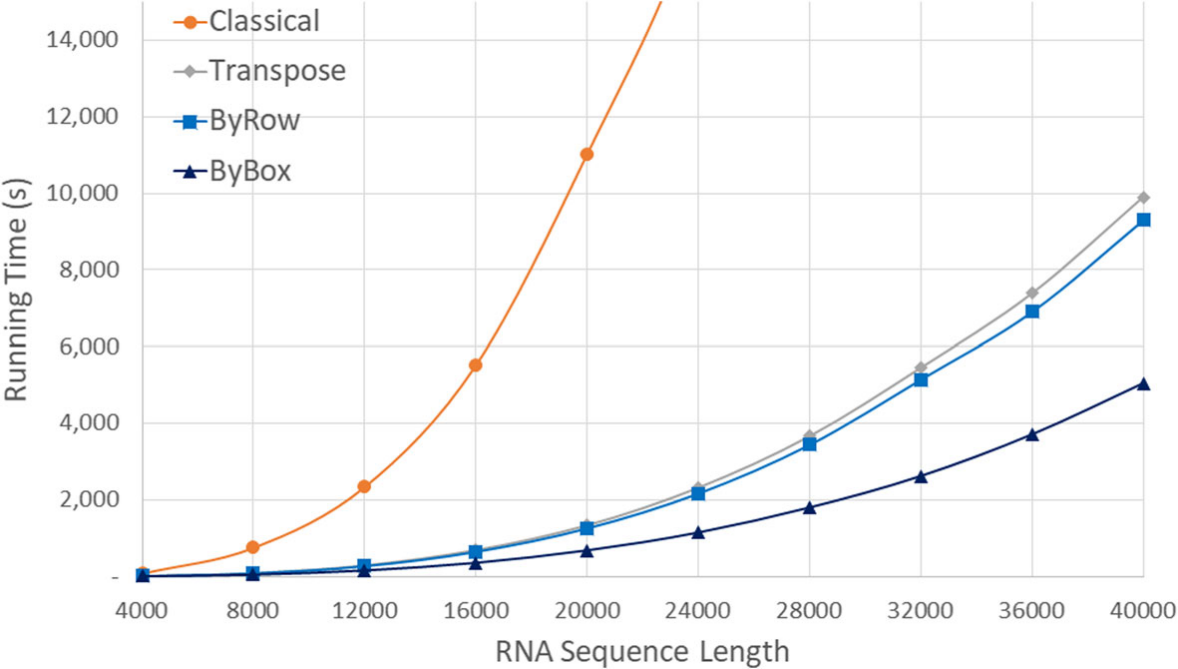
Run time, in seconds, for random sequences on Intel I7 platform

Figure 11 and Table 6 give the run times on our Intel I7 platform. Once again, we were unable to run *Classical* on our larger data sets (this time, *n*>28,000) because of the excessive time required by this algorithm on these larger data sets. As was the case for our Xeon and AMD platforms, the algorithms are ranked *ByBox*, *ByRow*, *Transpose*, *Classical*, fastest to slowest. The run time reduction achieved by *ByBox* is up to 93.70*%* relative to *Classical* and up to 51.92*%* relative to *Transpose*. *ByRow* is up to 89.19*%* faster than *Classical* and up to 15.62*%* faster than *Transpose*.

**Table 6:** Run time, in HH:mm:ss, for random sequences on Intel I7 platform

SeqLength	Classical	Transpose	ByRow	ByBox	RvsC	RvsT	BvsC	BvsT
4000	0:01:19	0:00:12	0:00:10	0:00:08	87.11%	15.62%	90.28%	36.35%
8000	0:12:33	0:01:30	0:01:21	0:00:51	89.19%	9.79%	93.19%	43.20%
12,000	0:38:58	0:04:56	0:04:33	0:02:40	88.31%	7.78%	93.17%	46.08%
16,000	1:31:52	0:11:36	0:10:48	0:06:00	88.24%	6.84%	93.46%	48.24%
20,000	3:03:31	0:22:23	0:21:01	0:11:25	88.55%	6.12%	93.78%	49.00%
24,000	4:52:55	0:38:45	0:36:11	0:19:12	87.65%	6.61%	93.45%	50.46%
28,000	7:56:51	1:01:12	0:57:23	0:30:03	87.97%	6.23%	93.70%	50.89%
32,000	-	1:30:58	1:25:48	0:43:44	-	5.68%	-	51.92%
36,000	-	2:03:10	1:55:06	1:01:52	-	6.54%	-	49.78%
40,000	-	2:45:08	2:35:03	1:24:07	-	6.10%	-	49.06%

#### Power PC A2

**Fig. 12:**
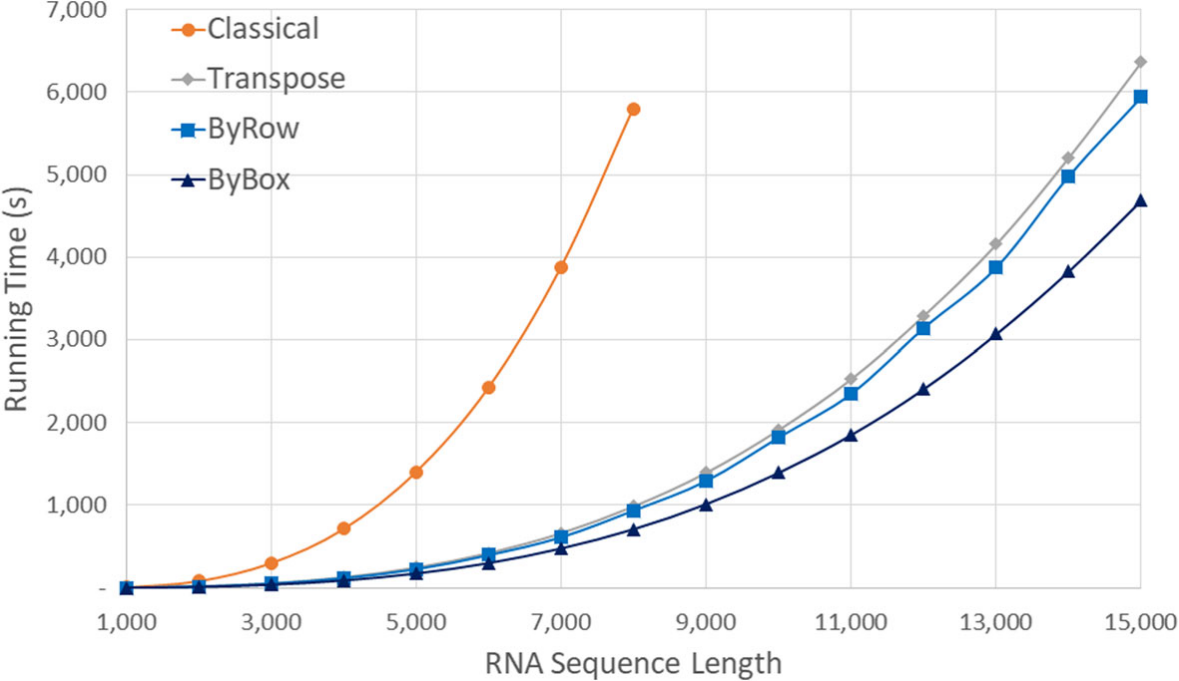
Run time, in seconds, for random sequences on the Power PC A2 platform

Figure 12 and Table 7 give the run times on our Power PC A2 platform. On this platform, we were able to run *Classical* only for *n*≤8000 and the remaining algorithms only for *n*≤15,000, because of the excessive time required by our algorithms on larger instances. On this platform, the speed ranking of our algorithms is consistent with our other 3 platforms. The ranking, fastest to slowest, is now *ByBox*, *ByRow*, *Transpose*, *Classical*. *ByBox* is up to 87.74*%* faster than *Classical* and up to 33.43*%* faster than *Transpose*, where *ByRow* is up to 84.18*%* faster than *Classical* and up to 14.68*%* faster than *Transpose*.

**Table 7:** Run time, in HH:mm:ss, for random sequences on the Power PC A2 platform

SeqLength	Classical	Transpose	ByRow	ByBox	RvsC	RvsT	BvsC	BvsT
1000	0:00:09	0:00:02	0:00:02	0:00:02	80.00%	14.68%	83.87%	31.19%
2000	0:01:27	0:00:17	0:00:15	0:00:11	82.99%	11.91%	87.14%	33.43%
3000	0:05:03	0:00:55	0:00:49	0:00:38	83.69%	9.64%	87.59%	31.25%
4000	0:11:55	0:02:08	0:01:58	0:01:29	83.52%	7.69%	87.57%	30.40%
5000	0:23:24	0:04:05	0:03:45	0:02:54	83.95%	8.06%	87.62%	29.08%
6000	0:40:24	0:07:01	0:06:36	0:05:00	83.65%	6.00%	87.62%	28.80%
7000	1:04:31	0:11:02	0:10:13	0:07:57	84.18%	7.43%	87.67%	27.90%
8000	1:36:36	0:16:27	0:15:36	0:11:50	83.85%	5.22%	87.74%	28.05%
9000		0:23:14	0:21:35	0:16:54		7.11%		27.26%
10,000		0:31:54	0:30:23	0:23:14		4.78%		27.17%
11,000		0:42:10	0:39:12	0:30:53		7.03%		26.75%
12,000		0:54:52	0:52:23	0:40:09		4.52%		26.82%
13,000		1:09:17	1:04:35	0:51:09		6.79%		26.18%
14,000		1:26:48	1:23:03	1:03:50		4.32%		26.46%
15,000		1:46:07	1:39:00	1:18:09		6.71%		26.34%

Table 8 gives the energy consumption in joules on our Power PC platform. As other platforms, the energy reduction by our cache efficient algorithms tracked run time quite closely. For example, while *ByBox* was almost always slower than *Transpose*, it almost always used less energy. *ByBox* reduced energy consumption by up to 87.59*%* relative to *Classical* and by up to 40.31*%* relative to *Transpose*. And *ByRow* is up to 82.6*%* and 16.7*%* relative to *Classical* and *Transpose*, respectively.

**Table 8:** Energy consumption, in joules, for random sequences on the Power PC A2

SeqLength	Original	Transpose	ByRow	ByBox	RvsO	RvsT	BvsO	BvsT
1000	30.77	8.17	6.80	4.97	77.89%	16.70%	83.86%	39.17%
2000	287.09	62.98	54.39	37.59	81.06%	13.65%	86.91%	40.31%
3000	999.30	203.04	180.26	125.87	81.96%	11.22%	87.40%	38.01%
4000	2,380.59	489.85	427.98	295.55	82.02%	12.63%	87.59%	39.67%
5000	4,632.91	931.05	824.09	583.93	82.21%	11.49%	87.40%	37.28%
6000	8,005.23	1,609.86	1,441.21	1,011.15	82.00%	10.48%	87.37%	37.19%
7000	12,822.30	2,510.05	2,226.54	1,615.56	82.64%	11.30%	87.40%	35.64%
8000	19,100.53	3,746.28	3,393.26	2,401.00	82.23%	9.42%	87.43%	35.91%
9000		5,310.33	4,709.12	3,430.15		11.32%		35.41%
10,000		7,290.13	6,647.03	4,709.83		8.82%		35.39%
11,000		9,434.29	8,569.82	6,276.69		9.16%		33.47%
12,000		12,606.74	11,375.63	7,994.03		9.77%		36.59%
13,000		15,121.14	14,056.36	10,291.69		7.04%		31.94%
14,000		19,849.26	18,233.34	12,840.73		8.14%		35.31%
15,000		24,308.08	21,648.41	15,661.49		10.94%		35.57%

### Java implementations

**Fig. 13:**
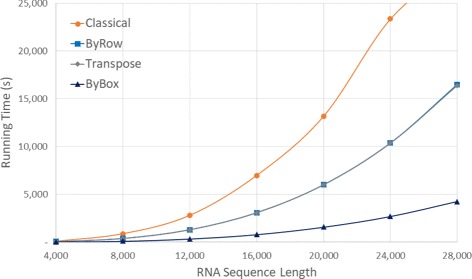
Run time, in seconds, for random sequences using our Java implementations on our Xeon E5 platform

**Fig. 14:**
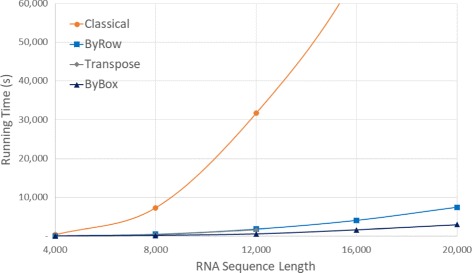
Run time, in seconds, for random sequences using our Java implementations on our AMD platform

**Fig. 15:**
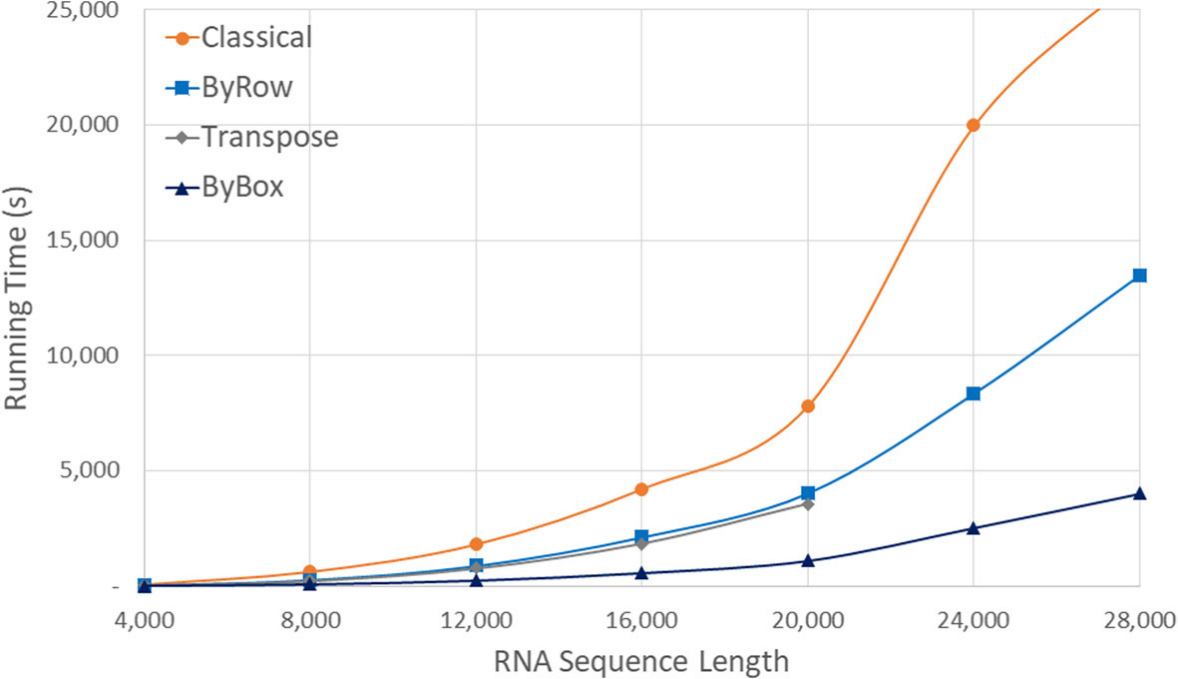
Run time, in seconds, for random sequences using our Java implementations on our Intel I7 platform

Figures 13, 14 and 15 and Tables 9, 10 and 11 give the run time for our Java implementations on our Xeon, AMD, and Intel platforms.

**Table 9:** Run time, in HH:mm:ss, for random sequences using our Java implementations on our Xeon E5 platform

SeqLength	Classical	Transpose	ByRow	ByBox	RvsC	RvsT	BvsC	BvsT
4000	0:01:29	0:00:49	0:00:49	0:00:13	45.18%	1.62%	85.72%	74.37%
8000	0:14:38	0:06:34	0:06:29	0:01:38	55.70%	1.30%	88.86%	75.17%
12,000	0:46:36	0:21:44	0:21:37	0:05:36	53.60%	0.55%	87.98%	74.23%
16,000	1:56:26	0:51:22	0:51:22	0:13:16	55.88%	-0.02%	88.60%	74.16%
20,000	3:39:36	1:40:09	1:40:05	0:26:10	54.42%	0.06%	88.08%	73.87%
24,000	6:29:22	2:52:43	2:52:54	0:44:43	55.59%	-0.11%	88.52%	74.11%
28,000	8:05:15	4:33:21	4:35:04	1:10:48	43.32%	-0.62%	85.41%	74.10%

**Table 10:** Run time, in HH:mm:ss, for random sequences using our Java implementations on our AMD platform

SeqLength	Classical	Transpose	ByRow	ByBox	RvsC	RvsT	BvsC	BvsT
4000	0:06:10	0:00:54	0:01:03	0:00:21	82.90%	-18.00%	94.23%	60.20%
8000	2:01:55	0:06:27	0:07:43	0:02:46	93.68%	-19.42%	97.73%	57.22%
12,000	8:48:01	0:26:02	0:29:56	0:09:12	94.33%	-14.97%	98.26%	64.64%
16,000	18:45:51	out of memory	1:07:26	0:26:48	94.01%		97.62%	
20,000	35:02:59	out of memory	2:04:00	0:48:39	94.10%		97.69%

**Table 11:** Run time, in HH:mm:ss, for random sequences using our Java implementations on our Intel I7 platform

SeqLength	Classical	Transpose	ByRow	ByBox	RvsC	RvsT	BvsC	BvsT
4000	0:01:01	0:00:29	0:00:34	0:00:09	44.54%	-16.54%	85.29%	69.10%
8000	0:10:12	0:03:51	0:04:31	0:01:11	55.79%	-17.19%	88.46%	69.41%
12,000	0:30:03	0:12:54	0:14:43	0:03:55	50.99%	-14.18%	86.94%	69.57%
16,000	1:10:05	0:30:44	0:35:27	0:09:19	49.40%	-15.36%	86.70%	69.67%
20,000	2:10:22	0:59:25	1:07:14	0:18:15	48.43%	-13.15%	86.00%	69.29%
24,000	5:33:02	out of memory	2:19:19	0:42:07	58.17%		87.35%	
28,000	7:22:37	out of memory	3:44:46	1:06:56	49.22%		84.88%	

The Java implementations take much substantially time and memory than do the C implementations. Because of memory limitations, *Transpose* could not be run on our AMD and Intel platforms for *n*≥16,000 and *n*≥24,000, respectively. Because of time requirements, we did not experiment with *n*>28,000 for any algorithm on any platform. The speed ranking, fastest to slowest, for the Java implementations is *ByBox*, *Transpose*, *ByRow*, *Classical*. The Java implementation of *ByBox* was up to 88.9*%* faster than the Java implementation of *Classical* on our Xeon platform, up to 98.3*%* faster on the AMD, and up to 88.5*%* faster on the Intel I7. The corresponding speedups relative to the Java implementation of *Transpose* were 75.2%, 64.6%, and 69.7%.

We observe that the run time of *ByRow* was generally more than that of *Transpose* on all of our platforms. We suspect this is because our Java code for *ByRow* makes more accesses to array elements than made by our Java code for *Transpose*. Array accesses are expensive in Java as the array indexes are checked for validity whenever an attempt is made to access an array element (we note that C does not perform such a check). Although some Java compilers eliminate this check when they can assert there will be no violation of array bounds, their ability to make this assertion is both variable and limited. In the case of *Transpose*, our code reduces the number of array accesses significantly by copying an array element that is to be used many times into a simple variable and then referring to this simple variable in reuses of the element. This reduction strategy could not be employed in the code for *ByRow*. As a result of the increased array bounds checking done in our Java code for *ByRow* relative to that done in our Java code for *Transpose*, the former is often slower.

## Discussion and conclusions

We have proposed three cache-efficient algorithms–*ByRow*, *ByRowSegment*, and *ByBox*–for RNA folding using Nussinov’s dynamic programming equations. Their cache miss efficiency was analyzed using a simple cache model. Although the simple cache model does not accurately reflect the cache architecture of modern computers, it is useful for an initial assessment of cache performance as the model encourages the design of algorithms with good spatial locality and good spatial locality results in better cache performance on virtually all cache architectures.

Our algorithms were benchmarked against the classical implementation, *Classical*, of Nussinov’s equations as well as the cache efficient implementation *Transpose* proposed by Li et al. [6]. The benchmarking was done using four different computational platforms (Xeon E5, AMD Athalon 64, Intel I7, Power PC A2) and two programming languages (C and Java). For the benchmarking, we excluded *ByRowSegement*, as, for the dataset sizes we could handle on our test platforms, *ByRow* and *ByRowSegment* are identical. Our benchmarking shows that, depending on the computational platform and programming language, either *ByRow* or *ByBox* give best run time and energy performance. In fact, the C version of these algorithms reduce run time by as much as 97.2*%* and energy consumption by as much as 88.8*%* relative to *Classical* and by as much as 56.3*%* and 57.8*%* relative to *Transpose*. The Java versions reduce run time by as much as 98.3% relative to *Classical* and by as much as 75.2% relative to *Transpose*.

The algorithms *ByRow*, *ByRowSegment*, *ByBox*, and *Classical* require about half as much memory as does *Transpose*. While run time becomes a limiting factor more often than memory, in our Java experiments, we were unable to run *Transpose* on our larger data sets on our AMD and Intel I7 platforms because of insufficient memory.

## Abbreviations

A: Adenine
AMD: Advanced micro devices
C: Cytosine
CPU: Central processing unit
DNA: DeoxyriboNucleic acid
GPU: Graphics processing unit
G: Guanine
LRU: Least recently used
LLC: Last level cache
NCBI: National center for biotechnology information
RNA: RiboNucleic acid
SW: Smith and waterman
U: Uracil
